# Molecular Classification of Breast Cancer Utilizing Long Non-Coding RNA (lncRNA) Transcriptomes Identifies Novel Diagnostic lncRNA Panel for Triple-Negative Breast Cancer

**DOI:** 10.3390/cancers13215350

**Published:** 2021-10-26

**Authors:** Hibah Shaath, Ramesh Elango, Nehad M. Alajez

**Affiliations:** 1 College of Health & Life Sciences, Hamad Bin Khalifa University (HBKU), Qatar Foundation (QF), Doha P.O. Box 34110, Qatar; hshaath@hbku.edu.qa; 2 Translational Cancer and Immunity Center (TCIC), Qatar Biomedical Research Institute (QBRI), Hamad Bin Khalifa University (HBKU), Qatar Foundation (QF), Doha P.O. Box 34110, Qatar; relango@hbku.edu.qa

**Keywords:** triple-negative breast cancer, TNBC, diagnosis, long non-coding RNA, lncRNA, gene signature

## Abstract

**Simple Summary:**

Breast cancer is the most commonly diagnosed cancer in women today and accounts for thousands of cancer-related deaths each year. While some breast cancer subtypes can be easily diagnosed and targeted for therapy, triple-negative breast cancer, which lacks receptor expression, is the most challenging to diagnose and treat. In this study, we use multiple RNA sequencing data to look specifically at long non-coding RNA (lncRNA) expression portraits at the transcript level and to identify lncRNA-based biomarkers associated with each breast cancer subtype. Receiver operating characteristic (ROC) analysis was used to validate their diagnostic potential, which was validated in two independent cohorts. Several lncRNA transcripts were found to be enriched in TNBC across all validation cohorts. Binary regression analysis identified a four lncRNA transcript signature with the highest diagnostic power for TNBC as potential novel biomarkers for diagnostic and therapeutic intervention. Interestingly, several of the identified lncRNAs were shown to have prognostic potential in TNBC.

**Abstract:**

Breast cancer remains the world’s most prevalent cancer, responsible for around 685,000 deaths globally despite international research efforts and advances in clinical management. While estrogen receptor positive (ER+), progesterone receptor positive (PR+), and human epidermal growth factor receptor positive (HER2+) subtypes are easily classified and can be targeted, there remains no direct diagnostic test for triple-negative breast cancer (TNBC), except for the lack of receptors expression. The identification of long non-coding RNAs (lncRNAs) and the roles they play in cancer progression has recently proven to be beneficial. In the current study, we utilize RNA sequencing data to identify lncRNA-based biomarkers associated with TNBC, ER+ subtypes, and normal breast tissue. The Marker Finder algorithm identified the lncRNA transcript panel most associated with each molecular subtype and the receiver operating characteristic (ROC) analysis was used to validate the diagnostic potential (area under the curve (AUC) of ≥8.0 and *p* value < 0.0001). Focusing on TNBC, findings from the discovery cohort were validated in an additional two cohorts, identifying 13 common lncRNA transcripts enriched in TNBC. Binary regression analysis identified a four lncRNA transcript signature (ENST00000425820.1, ENST00000448208.5, ENST00000521666.1, and ENST00000650510.1) with the highest diagnostic power for TNBC. The ENST00000671612.1 lncRNA transcript correlated with worse refractory free survival (RFS). Our data provides a step towards finding a novel diagnostic lncRNA-based panel for TNBC with potential therapeutic implications.

## 1. Introduction

Despite the global drive in breast cancer (BC) research and the remarkable advances in clinical management over recent years, released figures and estimates by the American Cancer Society for BC in the United States (US) for 2021 show the need for continued efforts in this field. The widespread incidence of BC continues, where in the US alone, 281,550 new cases of invasive breast cancer will be diagnosed in women (284,200 in men and women), and tens of thousands more non-invasive cases. Furthermore, 43,600 women and 530 men are predicted to lose their battles with BC this year in the US [1]. In 2020, there were 2.3 million women diagnosed with breast cancer and 685,000 deaths globally [2]. Such alarming statistics solidify the need for a deeper understanding of causes and mechanisms, thereby identifying potential diagnostic markers for more effective and personalized patient treatment plans.

The molecular classification of BC has been widely studied and commonly grouped into four categories based on hormone receptor expression: estrogen receptor positive (ER+); progesterone receptor positive (PR+); human epidermal growth factor receptor positive (HER2+); and triple-negative breast cancer (TNBC), which is characterized by the lack of expression of any of the mentioned receptors. This lack of expression in TNBC has remarkable implications on its diagnosis and treatment as it eliminates effective therapeutic targets (i.e., PR, ER, and HER2), causing TNBC to be the most aggressive BC subtype, highly metastatic and with overall poor survival rates in around 15% of all BC cases [3]. Hormone receptor positive (HR)/PR+ patients can be successfully treated with ER antagonists, such as tamoxifen, or by aromatase inhibitors [4], while HER+ BC patients are treated with antibodies or different molecules targeting the HER2 pathways, such as trastuzumab, pertuzumab, and lapatinib [5]. TNBC patients do not benefit from endocrine or HER2-targeted therapies; therefore, chemotherapy and surgery, which can be highly invasive, remain the main treatment modality for those patients [6].

Neoadjuvant chemotherapy (NAC) is mainly given to facilitate breast-conserving surgery (BCS) and to eliminate clinically silent micro-metastases, or aid in downsizing tumors when patients are considered inoperable [7]. Clinical trials have shown that patients’ breast tumor pathologic complete response (pCR) to NAC was significantly higher in those with triple-negative tumors and HER2-positive tumors (47.9% and 50.2%, respectively) than in those with hormone receptor positive, HER2-negative tumors (15.5%, *p* < 0.0001) [8]. Although the initial response has been encouraging in comparison to other BC subtypes [9,10,11], recurrence and death rates were higher for TNBC in the first 3 years, and patients with residual disease (RD), which is still over 50%, had worse overall survival (OS) rates if they had TNBC compared with non-TNBC patients (*p* < 0001) [12]. Finding alternative approaches to diagnose and treat TNBC remains an important aspiration. Recent findings on the significance of long non-coding RNAs (lncRNAs) in many biological processes have demonstrated their potential function in modulating TNBC, including tumor-suppressor and oncogenic pathways that may serve as prognostic markers in BC [13].

In recent years, lncRNAs have been identified as playing an important role in various biological process, and their differential expression was implicated in numerous cancer types, including breast [14], lung adenocarcinoma [15], gastric [16], and leukemia [17]. Metastasis-associated lung adenocarcinoma transcript 1 (MALAT1) is a well-known lncRNA, shown to be either downregulated or upregulated in different types of cancers [18,19]. Several lncRNAs, including HOTAIR, ANRIL, ZFAS1, HOTAIRM1, PVT1, and LNP1, are associated with BC [20,21]. Tumor suppressor lncRNAs, such as GAS5, OPORS-AS1 and XIST, and oncogenic lncRNAs, namely H19, SRA, LSINCT5, Zfas1, Smad7, LOC554202, HOTAIR, SOX2OT, and FAL1, have been reported in BC [22]. Our previous study identified LINC01614 to be enriched in the luminalB/HER2+ subtype [13].

In the present study, we employed computational analysis to characterize the lncRNA transcriptome in a cohort of TNBC, ER+, and normal breast tissue. The marker finder algorithm was subsequently employed to identify lncRNA transcripts distinctive of each molecular subtype. Top identified markers were subsequently subjected to receiver operating characteristic (ROC) analysis to validate their diagnostic potential and to further refine the lncRNA panels associated with each molecular subtype, and their prognostic value was further assessed. Our findings can have an impact on our understanding of the role of lncRNAs in TNBC and their potential utilization in diagnosis and therapeutic intervention methods, which warrant further investigation.

## 2. Materials and Methods

### 2.1. RNA-Seq Data Analysis

Raw RNA sequencing data were retrieved from the sequence read archive (SRA) database under accession no. PRJNA251383, consisting of 42 TNBC; 42 ER+; and 56 normal breast tissue samples. FASTQ files were subsequently mapped and aligned to the hg38 ncRNA (non-coding RNAs) assembly using KALLISTO 0.4.2.1, as previously described [23,24]. Normalized expression data (TPM (transcripts per million)) mapped reads were sequentially imported into the AltAnalyze v.2.1.3 software for differential expression analysis using 2.0-fold change and adjusted <0.05 *p* value cut-off. Transcripts were excluded from an analysis based on TPM (<1.0 raw expression value). The Benjamini–Hochberg method was used to adjust for false discovery rate (FDR). The marker finder prediction was carried out as previously explained [23,25]. The marker finder analysis was achieved within each experimental condition to predict specific markers based on enrichment. The PRJNA486023 (360 TNBC and 88 normal) and PRJNA553096 (72 TNBC and 19 normal) were used as validation cohorts.

### 2.2. ROC and Binary Regression Analysis

ROC analysis was subsequently used to assess the accuracy of model predictions by plotting the sensitivity versus the 1-specificity of a classification test (as the threshold varies over an entire range of diagnostic test results). The full area under a given ROC curve, or AUC, formulates an important statistic that represents the probability that the prediction will be in the correct order when a test variable is observed. The ROC analysis was conducted in SPSS version 26 (IBM-SPSS Inc., Chicago, IL, USA), and area AUC of >0.8 and *p* < 0.0001 were considered significant. Binary regression analysis for the best predictors of TNBC was constructed in SPSS 26.

### 2.3. Survival and Statistical Analysis

Kaplan–Meier survival analysis and plotting were conducted using IBM SPSS version 26 software. For survival analysis, patients were grouped into high or low based on the corresponding lncRNA median expression. The log-rank test was used to compare the outcome between expression groups. Graphpad Prism 9.0 software (San Diego, CA, USA) was used to compare the lncRNA expression as a function of tumor grade and LN status. An unpaired two-tailed t-test was used to compare two groups, while a one-way Anova was used to compare multiple groups. The *p* value of <0.05 was considered as significant.

## 3. Results

### 3.1. Identification of lncRNA-Based Biomarkers Associated with TNBC, ER+, and Normal Breast Tissue

**Figure 1:**
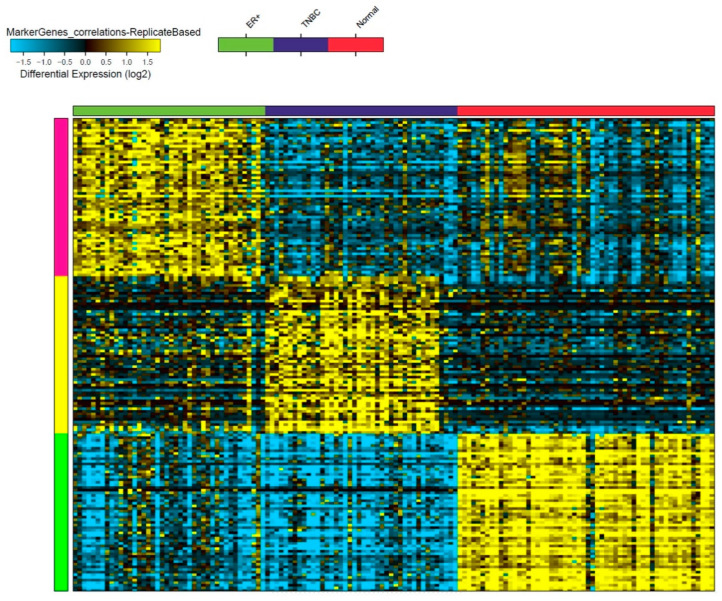
Identification of lncRNA-based biomarkers associated with TNBC, ER+, and normal breast tissue. Heatmap image depicting putative lncRNA-based markers associated with TNBC, ER+, and normal breast tissue, employing the marker discovery algorithm. Each column represents a sample while each row represents an lncRNA transcript. The first block of samples shown under the green x axis represents ER+ samples, the purple represents the TNBC samples, and the red represents the normal breast tissue samples. The expression of each lncRNA transcript is depicted according to the color scale (blue = downregulation and yellow = upregulation, differential expression (log2)).

Raw RNA sequencing data from 42 TNBC, 42 ER+, and 56 normal breast tissue samples were retrieved from the sequence read archive (SRA) database under accession no. PRJNA251383, and a total of 57,942 ncRNA transcripts were analyzed using the Kallisto v0.4.2.1 algorithm. The marker finder algorithm was then used to identify the sets of lncRNA transcripts distinctive of each molecular subtype (TNBC, ER+, and normal) (Figure 1). Our data revealed three clusters based on the lncRNA expression (TNBC, ER+, and normal clusters). The list of the top 60 lncRNA indicative of each molecular subtype is detailed in supplementary Table S1. Notably, we observed 15 of the 60 enriched lncRNA transcripts in TNBC to belong to the *LINC00511* gene.

### 3.2. Receiver Operating Characteristic (ROC) Curves for Putative lncRNA Markers Associated with TNBC, ER+, and Normal Breast Tissue

**Figure 2:**
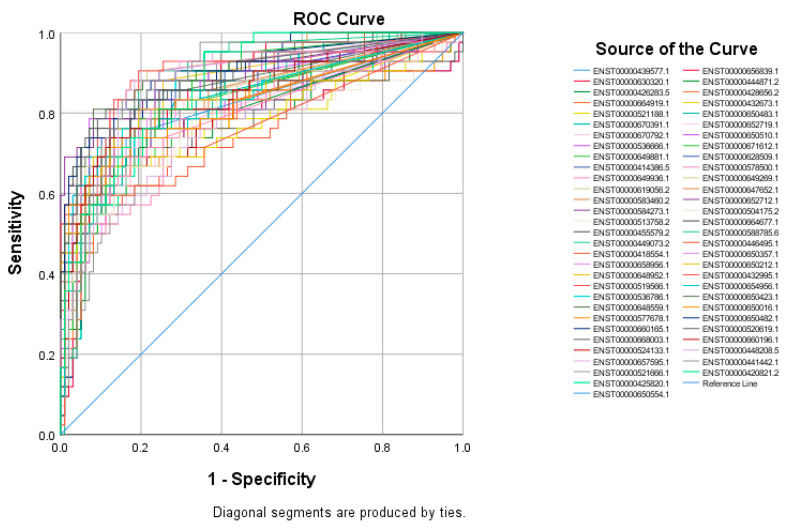
ROC curves for putative lncRNA markers associated with TNBC. The top sixty identified lncRNA markers for TNBC using the marker finder algorithm were subjected to ROC analysis in SPSS. LncRNAs exhibiting an area under the curve of >0.8 and *p* < 0.0001 are included.

**Figure 3:**
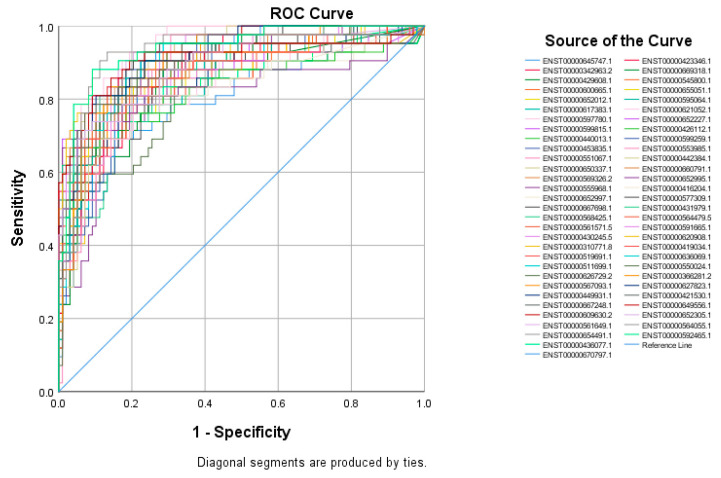
ROC curves for putative lncRNA markers associated with ER+ BC. The top sixty identified lncRNA markers for ER+ using the marker finder algorithm were subjected to ROC analysis in SPSS. LncRNAs exhibiting an area under the curve of >0.8 and *p* < 0.0001 are included.

**Figure 4:**
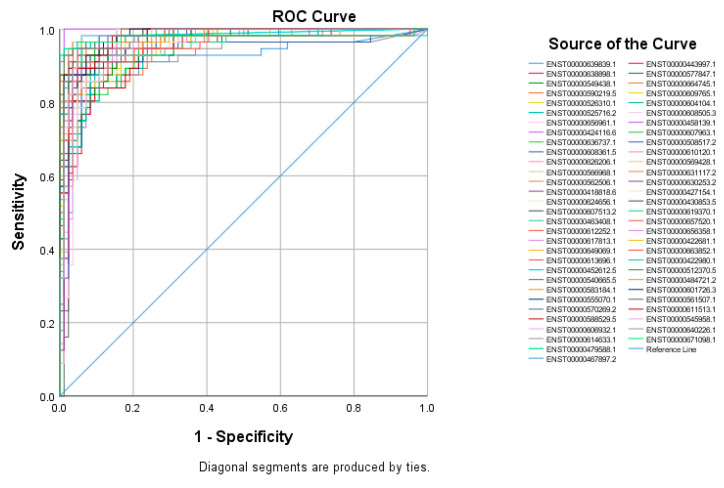
ROC curves for putative lncRNA markers associated with normal breast tissue. The top sixty identified lncRNA markers for normal breast tissues from the marker finder algorithm were subjected to ROC analysis in SPSS. LncRNAs exhibiting an area under the curve of >0.8 and *p* < 0.0001 are included.

The top 60 identified lncRNAs for TNBC, ER+, and normal breast tissue were subsequently subjected to ROC test analysis to validate their diagnostic potential and to identify the lncRNA panel most associated with each molecular subtype. LncRNA transcripts which exhibited areas under the curve (AUC) of ≥8.0 and an asymptotic *p* value < 0.0001 were included in the models. The ROC analysis for putative lncRNA markers associated with TNBC identified 47 lncRNA transcripts, which fulfilled the aforementioned criteria (Figure 2). Similarly, ROC analysis confirmed the diagnostic potential of the top 60 identified lncRNA transcripts for ER+ BC and normal breast tissue identified using the marker finder algorithm (Figure 3 and Figure 4, respectively). The list of identified lncRNA transcripts, AUC, and associated *p* values are provided in supplementary Tables S2–S4.

### 3.3. Validation of Common lncRNA Markers in the Second Cohort of TNBC and Normal Breast Tissue

**Figure 5:**
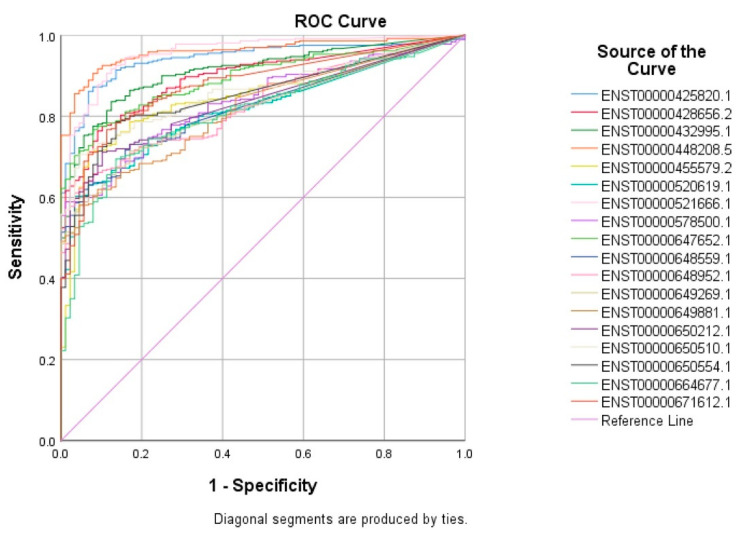
The validation of 18 common lncRNA markers in the second cohort of 360 TNBC samples and 88 normal breast tissue samples. A total of forty seven lncRNA transcripts identified from the marker finder algorithm were then validated in a second cohort of 360 TNBC samples and 88 normal breast tissue samples. LncRNAs exhibiting the values of >0.8 AUC and *p* < 0.0001 were retained.

Given that TNBC currently has no positive diagnostic markers, we focused the remaining part of the study on TNBC. The identified 47 lncRNA transcripts exhibiting > 0.8 AUC were then validated in a second cohort of 360 TNBC samples and 88 normal breast tissue samples (accession no. PRJNA486023), which identified 18 common lncRNA transcripts with a high diagnostic potential (AUC ≥ 8.0 and an asymptotic *p* value of <0.0001), as shown in Figure 5 and the supplementary Table S5.

**Figure 6:**
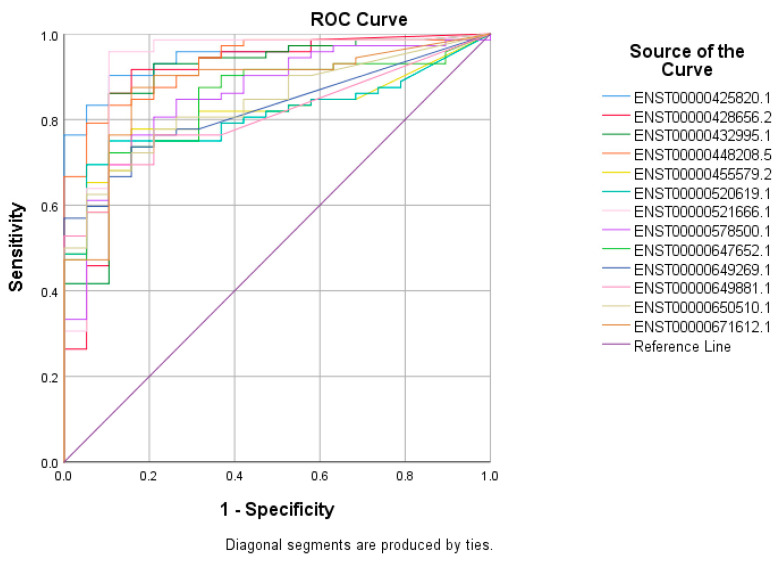
The validation of 13 common lncRNA markers in a third cohort of 72 TNBC samples and 19 normal breast tissue samples. A total pf eighteen lncRNA transcripts identified from the marker finder algorithm which showed the values of >0.8 AUC and *p* < 0.0001 were then validated in a third cohort of 72 TNBC samples and 19 normal breast tissue samples, and the lncRNAs exhibiting the values of >0.8 AUC and *p* < 0.0001 were retained.

The 18 identified TNBC-specific lncRNA transcripts were then validated in a third cohort (accession on. PRJNA553096) of 72 TNBC samples and 19 normal breast tissue samples, which further identified 13 lncRNA transcripts, namely ENST00000425820.1; ENST00000428656.2; ENST00000432995.1; ENST00000448208.5; ENST00000455579.2; ENST00000520619.1; ENST00000521666.1; ENST00000578500.1; ENST00000647652.1; ENST00000649269.1; ENST00000649881.1; ENST00000650510.1; and ENST00000671612.1, which were common and validated across the three cohorts (Figure 6 and supplementary Table S6).

**Figure 7:**
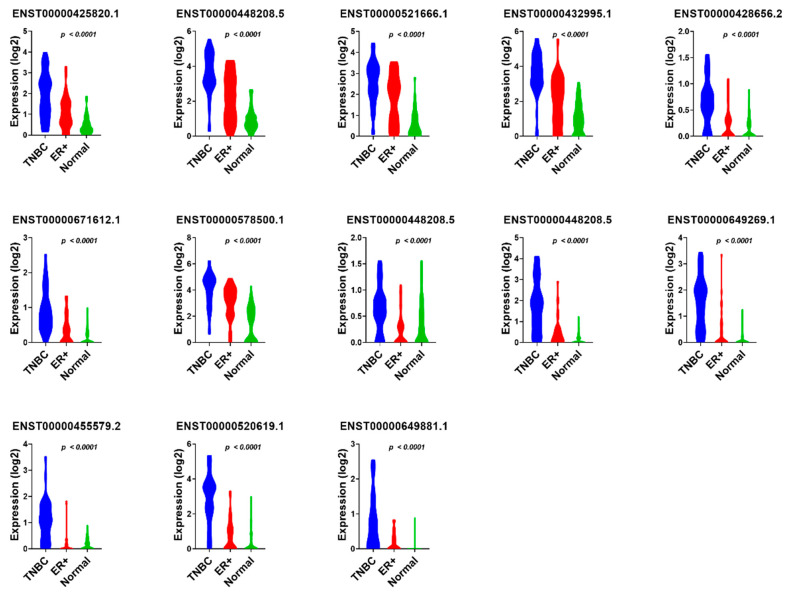
The expression value of 13 TNBC diagnostic lncRNA panels in TNBC compared to ER+ and normal breast tissue samples from the PRJNA251383 cohort. The expression values (log2) of TNBC (*n* = 42), ER+ (*n* = 42), and normal (*n* = 56) from the discovery cohort are presented as violin plots with the Anova *p* value indicated on each plot.

**Figure 8:**
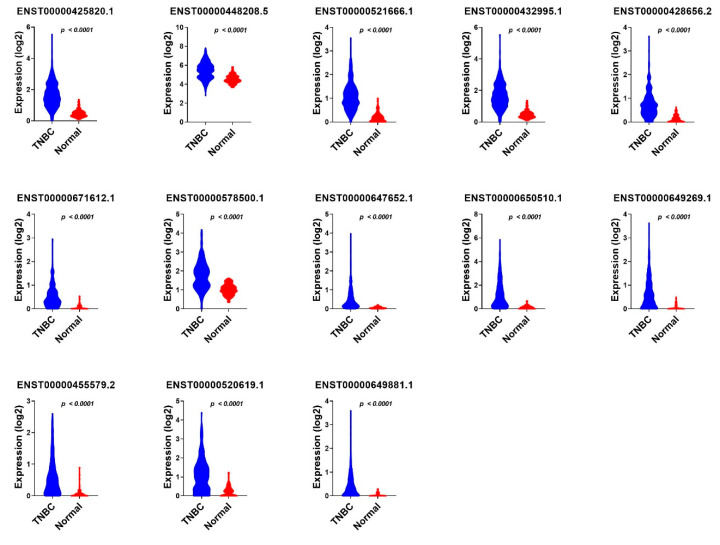
The expression value of 13 TNBC diagnostic lncRNA panels in TNBC compared to normal breast tissue samples from the PRJNA486023 cohort. The expression values (log2) of TNBC (*n* = 360) and normal (*n* = 88) from the first validation cohort are presented as violin plots with the Anova *p* value indicated on each plot.

**Figure 9:**
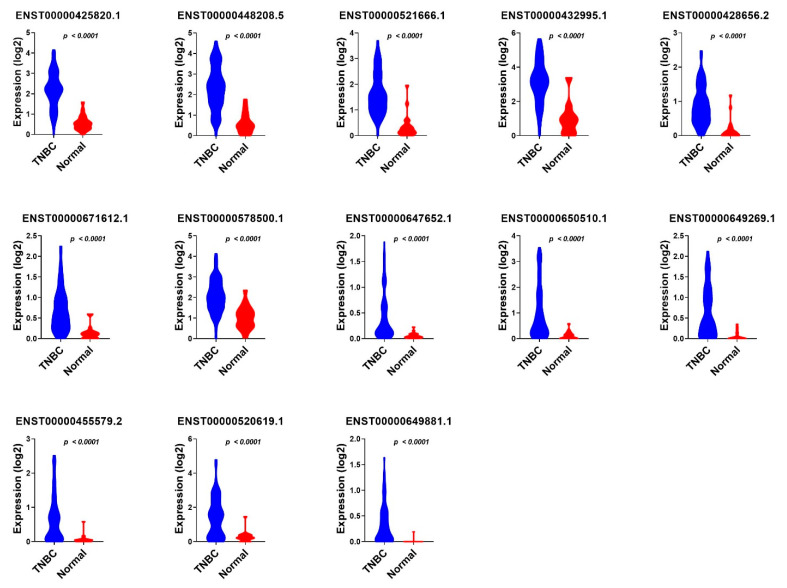
The expression of 13 TNBC diagnostic lncRNA panels in TNBC compared to normal breast tissue samples from the PRJNA553096 cohort. The expression values (log2) from TNBC (*n* = 72) and normal (*n* = 19) from the second validation cohort are presented as violin plots with the Anova *p* value indicated on each plot.

We subsequently sought to determine if the combination of those 13 lncRNA transcripts have a higher predictive power; therefore, the 13 identified lncRNA transcripts were subjected to binary regression analysis (forward LR) in SPSS 26, which identified a combination of four lncRNA transcripts (ENST00000448208.5; ENST00000521666.1; ENST00000650510.1; and ENST00000425820.1) with the highest diagnostic power for TNBC (Table 1). The expression of the final 13 identified diagnostic lncRNA transcripts in TNBC, ER+, and normal breast tissue samples from the PRJNA251383 cohort is shown in Figure 7, while the expression of the same set of lncRNA transcripts in TNBC compared to normal breast tissue samples from the PRJNA486023 and PRJNA553096 cohorts is presented in Figure 8 and Figure 9, respectively.

**Figure 10:**
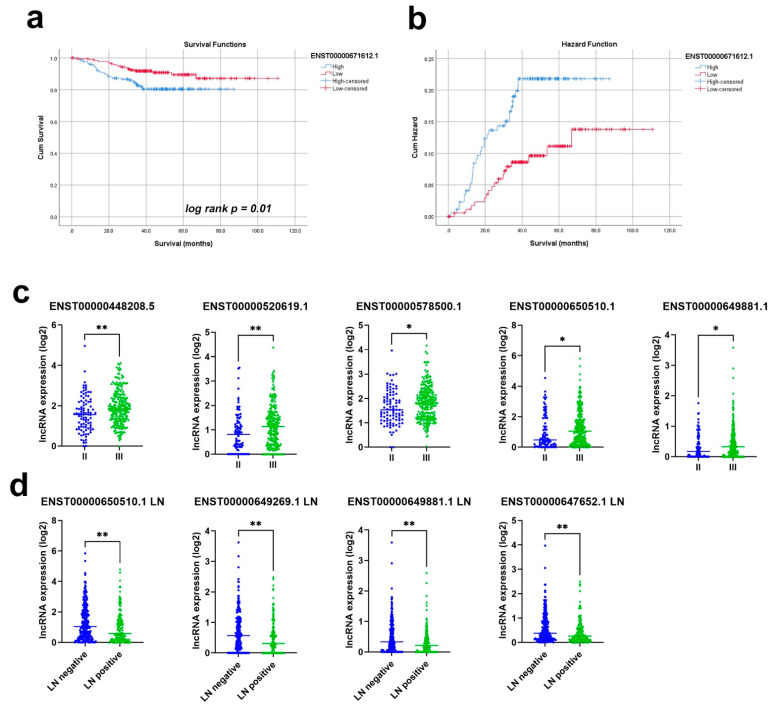
Prognostic value of the identified thirteen lncRNA panels in TNBC. The Kaplan–Meier survival (**a**) and hazard (**b**) analysis based on the ENST00000671612.1 expression. (**c**) The expression of ENST00000448208.5; ENST00000520619.1; ENST00000578500.1; ENST00000650510.1; and ENST00000649881.1 in TNBC, with grade II compared to grade III. (**d**) The expression of ENST00000650510.1, ENST00000649269.1, ENST00000649881.1, and ENST00000647652.1 in TNBC patients with and without LN involvement. * *p* < 0.05, ** *p* < 0.005.

We subsequently assessed the prognostic power of the 13 identified lncRNA transcripts in a cohort of 360 TNBC patients (PRJNA486023). The cohort was divided into high and low based on the median lncRNA expression and was subsequently subjected to the Kaplan–Meier survival analysis. Among the thirteen lncRNAs, ENST00000671612.1 correlated with worse refractory free survival (RFS); *p* value = 0.01; and HR = 2.0 (1.1–3.7), as presented in Figure 10a,b, while ENST00000520619.1 exhibited marginal significance in predicting RFS (*p* = 0.15, supplementary Figure S1). When patients were divided according to tumor grade II vs. III, ENST00000448208.5; ENST00000520619.1; ENST00000578500.1′ ENST00000650510.1′ and ENST00000649881.1 exhibited a higher expression in grade III tumors (Figure 10c). On the other hand, the expression of ENST00000650510.1, ENST00000649269.1, ENST00000649881.1, and ENST00000647652.1 was lower in patients with lymph node metastasis compared to those without metastasis (Figure 10d). Taken together, our data highlighted the plausible prognostic role of those lncRNAs in TNBC.

## 4. Discussion

Research into alternative methods of diagnosis and treatment in combination with the available chemotherapies and immunotherapies, or as stand-alone methods, are required to increase the effectiveness of individual treatment plans, leading to more comfortable modes of therapy, better pCR, and the overall survival and quality of life for TNBC patients. Several studies have previously explored potential protein coding signatures for TNBC prognosis, suggesting the ability to distinguish between subsets of BC patients to permit tailored therapeutic regimens based on risk [26], physiological variations [27], or NAC resistance [28]. In the context of ncRNAs, our recent findings implicated TGFβ signaling; a major player in cancer development, in shaping the lncRNA and miRNA transcriptomes of TNBC [29]. Using single cell transcriptome and functional validation, we recently implicated MALAT1 in TNBC resistance to NAC [30].

While the majority of published literature explored the diagnostic and prognostic potential of protein coding transcriptomes in BC, in the current study, we utilized computational analysis and multiple RNA-Seq data sets and identified a panel of 13 lncRNA transcripts enriched in TNBC, which was validated in two independent cohorts consisting of 432 TNBC and 107 normal breast tissue samples. Moreover, our data also highlighted a prognostic value for several of the identified lncRNA transcripts in TNBC.

Interestingly, fifteen of the sixty lncRNA transcripts enriched in TNBC based on our initial analysis belong to the LINC00511 family, seven of which made it to the final thirteen candidates when validated across the additional two cohorts. LncRNA LINC00511 has been characterized in several cancer types. In agreement with our data, Zhang et al. reported the elevated expression of *LINC00511* in ER-negative BC, which correlated with a poor prognosis. Functional studies knocking down the expression of *LINC00511* revealed significant inhibitory effects on the viability and proliferation of BC cell lines. In addition to this, the overexpression of *LINC00511* substantially promoted its viability and proliferation by accelerating the G1/S transition through the downstream regulation of the expression of CDKN1B [31]. However, our data precisely identified the specific transcripts which were associated with TNBC. In gastric cancer (GC), *LINC00511* was highly expressed and further studies found LINC00511 to recruit EZH2 (enhancer of zeste 2 polycomb repressive complex 2 subunit) to the *PTEN* (phosphatase and tensin homolog) promoter, facilitating its methylation, and subsequently activating the PI3K/AKT pathway, promoting GC cell proliferation, migration, and stemness, while inhibiting GC cell apoptosis [32]. LINC00511 was also found to regulate the expression of microRNA-625-5p and activate signal transducers and activators of transcription 3 (STAT3) to accelerate the progression of GC [33].

In colorectal cancer (CRC), the overexpression of *LINC00511* accelerated CRC development by facilitating cell proliferation, metastasis, and stemness. Studies found that LINC00511 acted as a competing endogenous RNA (ceRNA) with NFIA (Nuclear Factor I A) to bind with miR-29c-3p, revealing the LINC00511/miR-29c-3p/NFIA axis as potential therapeutic targets for CRC treatment [33,34]. In a study on temozolomide (TMZ), the first-line chemotherapy drug for glioblastoma (GBM), *LINC00511* expression upregulation correlated with the poor prognosis of GBM patients and *LINC00511* silencing impaired the tolerance of TMZ, while its overexpression increased the TMZ resistance of sensitive GBM cells [35]. In addition to this, the silencing of *LINC00511* expression suppressed cell viability, proliferation, migration, and invasion, and accelerated the apoptosis of glioma cells via a suggested miR-15a-5p/AEBP1 axis [36]. Other studies have associated LINC00511 with breast cancer [37], hepatocellular carcinoma [38], and bladder carcinoma [39].

A further three transcripts in the final thirteen TNBC-specific lncRNAs are antisense transcripts of diaphanous-related formin 3 (DIAPH3). In hepatocellular carcinoma [40], lung adenocarcinoma [41], and pancreatic cancer [42], the oncogenic roles played *by DIAPH3* expression have all been described. In pancreatic cancer, DIAPH3 promoted the proliferation, anchorage-independent growth, and invasion of cancer cells by the activation of selenoprotein TrxR1-mediated antioxidant effects [42]. In lung adenocarcinomas, the knockdown of *DIAPH3* inhibited tumorigenesis in both nude mice and in de novo mouse models via impaired ERK signaling [41]. In hepatocellular carcinoma, *DIAPH3* expression activated beta-catenin/TCF signaling by binding HSP90 and disrupting the interaction between GSK3 beta and HSP90 [40]. In TNBC, *DIAPH3* expression was associated with TNM stage and lymph node metastasis, but not with tumor size in patients [43]. While those studies highlighted the oncogenic role of DAIPH3 in several cancer types, the roles of the identified DIAPH3 antisense lncRNA transcripts in our study still warrant further investigation. Alternatively, DIAPH3 antisense transcripts can be co-expressed with DIAPH3 due to a shared promoter, which remains to be investigated. Interestingly, one of the final thirteen TNBC-specific lncRNAs encoding DIAPH3 antisense RNA 1 (or ENST00000671612.1) correlated with worse RFS when subjected to the Kaplan–Meier survival analysis, *p* value = 0.01, and HR = 2.0 (1.1–3.7). Transcript ENST00000520619.1, which exhibited a marginal significance in predicting RFS in our analysis (*p* = 0.15, supplementary Figure S1), also known as small nucleolar RNA host gene 6 (SNHG6), has recently been proposed as a therapeutic target for TNBC via the modulation of the miR-125b-5p/BMPR1B axis. *SNHG6* knockdown inhibited TNBC cell proliferation and migration, while promoting cell apoptosis. Suppressed *SNHG6* also resulted in lower tumor weights and volumes in xenograft mouse models, thus supporting our findings [44].

The four lncRNA transcripts most highly diagnostic for TNBC in our study were ENST00000448208.5, ENST00000521666.1, ENST00000650510.1, and ENST00000425820.1. ENST00000448208.5 is a transcript for the *SGO1-AS1* gene encoding the antisense of the SGO1 protein, a member of the shugoshin family of proteins that protects the centromere during mitosis, with regards to spindle assembly [45]. SGO1-AS1 has been recently connected to several cancers, including colorectal [46] and breast cancer. In breast cancer, a study on 39 breast cancer tissue samples reported that SGO1-AS1 was considerably down regulated in tumor tissues compared with adjacent non-cancer tissues, and transcript quantities of SGO1-AS1 were associated with age at the onset of the disease (*p* = 0.01). The expression of *SGO1*, on the other hand, presented no significant differences between tumor and non-tumor tissues [47]. In colorectal cancer, 40 tumor tissue samples were studied against normal adjacent tissue samples and suggested a significant decrease in SGO1 in colorectal cancer tumor samples (*p* < 0.001), and SGO1-AS1 lncRNA was significantly upregulated, compared to adjacent healthy tissues, clearly distinguishing the two populations [46]. Of the two articles on SGO1-AS1 published to date, one study was concordant with our results and the other showed opposing findings. This could be due to the fact that our study looks at the individual transcript expression levels as opposed to the gene expression levels, which could attribute to the differences in the reported literature. In addition to this, SGO1-AS1 was found to have a higher expression in grade III tumors compared to grade II tumors when patients were divided according to tumor grades. A more in-depth analysis of transcript expression levels provides further accuracy and clarification of specific potential biomarkers, warranting further investigation and functional studies into the precise effects of these lncRNAs in TNBC.

ENST00000521666.1, a hyaluronan mediated motility receptor antisense RNA 1 (HMMR-AS1) transcript codes the antisense for HMMR, which is implicated with adverse tumor progression via the TGF-beta/Smad2 signaling pathway [48,49]. HMMR-AS1 has been associated with glioblastoma (GBM), ovarian cancer, lung adenocarcinoma (LUAD), and basal-like breast cancer cells [48,49,50,51,52,53]. *HMMR-AS1* is described as hyper-expressed in GBM cell lines, in which its knockdown reduces *HMMR* expression inhibiting cell migration, invasion, and mesenchymal phenotypes, suppressing GBM cell growth in both in vitro and in vivo experiments [50]. In ovarian cancer, *HMMR-AS1* was significantly upregulated in tumor tissues compared to normal tissues, which was related to advanced stages and lymphatic metastasis with a shorter overall survival time (*p* = 0.0075), and progression-free survival time (*p* = 0.0013) than those with lower *HMMR-AS1* expression [51]. A significant upregulation of *HMMR-AS1* detected in LUAD was also associated with a larger tumor diameter; an advanced TNM stage, lymph node metastasis, and a shorter survival time, where its inhibition reduced tumor progression and metastasis [52]. In another study, the proliferation and migration abilities of the cell lines MDA-MB-231 and MDA-MB-468 TNBC were suppressed after the knockdown of HMMR-AS1 in vitro [53].

ENST00000650510.1 is one of many *LINC00511* lncRNA transcripts (LINC00511-301). While this transcript has not been reported on specifically, several studies have associated LINC00511 with different cancer types, as previously discussed. LINC00511-301 was also identified in our study to be associated with tumor grade III. When patients were divided according to tumor grade II vs. III, LINC00511-301 exhibited a higher expression in grade III tumors, but was lower in patients with lymph node metastasis compared to those without metastasis. Another *LINC00511* lncRNA from the final 13, ENST00000649881.1 or LINC00511-276, was also highly expressed in grade III tumors compared to grade II tumors. In addition, ENST00000649269.1 and ENST00000649881.1, or LINC00511-256 and LINC00511-276, respectively, were lower in patients with lymph node metastasis compared to those without metastasis. Further studies focusing on the effects of these particular transcripts are important to understand the precise role they play in the potential prognosis and diagnosis of TNBC. Finally, ENST00000425820.1, the last of the four most highly diagnostic transcripts for TNBC, is a novel transcript encoding an lncRNA overlapping with the DEPDC1 protein coding gene; therefore, the role of this novel lncRNA in TNBC remains to be investigated.

Developing our understanding of the precise involvement of lncRNAs in biological processes can indicate their use as targets in BC therapies. In a study by Battistelli et al., the function of a master EMT-transcription factor was effectively impaired after a *HOTAIR* deletion mutant, including the putative snail-binding domain but depleted of the EZH2-binding domain, acted as a dominant negative of the endogenous *HOTAIR*. This mutant form was subsequently able to reduce cellular motility, invasiveness, anchorage-independent growth, and responsiveness to TGFβ-induced EMT [54]. Studies focusing on manipulating and translating such dominant competitiveness of deletion mutants can propel novel strategies into RNA-based therapeutic options. Multiple oligonucleotide drugs have been approved and a dozen more are in phase III trials, primarily for genetically well-defined rare diseases [55]; however, the current focus on RNA therapeutics has the potential to provide further clinical success across other disease types, including cancer.

Currently there is no specific diagnostic test for TNBC except for the lack of expression of HER2 and hormone receptors (ER and PR). In hindsight, such studies can successfully decipher signatures with predictive potential; however, signatures of diagnostic potential are in great need and will impact the accurate diagnosis and treatment of diseases, particularly for TNBC subtypes. Whether the outcomes of this study can aid in diagnosis for clinical potential or for therapeutic application currently remains a prospect. Additional validation in larger cohorts side by side, compared to current diagnostic and prognostic panels for TNBC, are needed to assess the validity of identified lncRNA panels from the current study.

## 5. Conclusions

Our presented data confirms the validity of the analysis used in our current study and solidifies the need for further clinical studies on the feasibility of the identified lncRNA candidates as potential diagnostic markers, specifically for TNBC. From our data, we identified four lncRNAs: SGO1-AS1, HMMR-AS1, LINC00511, and transcript ENST00000650510.1, two of which have limited studies in TNBC and two that are novel. This research also highlights the association of prognostic values, tumor stages, and lymph node metastasis with aberrant lncRNA transcript expression levels, taking a step towards finding a novel diagnostic lncRNA-based panel for TNBC, while their potential utilization in diagnosis, prognosis, or therapeutic targets requires further investigation.

## Figures and Tables

**Table 1:** Binary regression analysis (forward LR) in SPSS 26.

		B	S.E.	Wald	df	Sig.	Exp(B)	95% C.I. for EXP(B)	
								Lower	Upper
Step 1 ^a^	ENST00000448208.5	5.227	0.636	67.531	1	0.000	186.228	53.534	647.832
	Constant	−2.794	0.400	48.786	1	0.000	0.061		
Step 2 ^b^	ENST00000448208.5	3.516	0.711	24.418	1	0.000	33.643	8.342	135.681
	ENST00000521666.1	4.179	0.914	20.906	1	0.000	65.290	10.887	391.561
	Constant	−3.367	0.466	52.301	1	0.000	0.034		
Step 3 ^c^	ENST00000448208.5	3.392	0.764	19.704	1	0.000	29.725	6.648	132.912
	ENST00000521666.1	4.198	0.935	20.153	1	0.000	66.557	10.646	416.090
	ENST00000650510.1	3.222	0.983	10.743	1	0.001	25.069	3.651	172.103
	Constant	−4.074	0.563	52.441	1	0.000	0.017		
Step 4 ^d^	ENST00000425820.1	1.262	0.587	4.622	1	0.032	3.533	1.118	11.166
	ENST00000448208.5	3.146	0.836	14.166	1	0.000	23.242	4.516	119.606
	ENST00000521666.1	3.085	1.036	8.861	1	0.003	21.858	2.868	166.574
	ENST00000650510.1	3.338	0.983	11.525	1	0.001	28.175	4.100	193.615
	Constant	−4.498	0.617	53.132	1	0.000	0.011		

^a^ Variable(s) entered in step 1: ENST00000448208.5.; ^b^ variable(s) entered in step 2: ENST00000521666.1.; ^c^ variable(s) entered in step 3: ENST00000650510.1.; and ^d^ variable(s) entered in step 4: ENST00000425820.1.

## Data Availability

The data presented in this study are available in this article and supplementary material.

## Supplementary Materials

The following materials are available online at https://www.mdpi.com/article/10.3390/cancers13215350/s1; Table S1: LncRNA transcripts predicative of the indicated molecular subtype; Table S2: ROC analysis for 60 lncRNA transcripts associated with TNBC; Table S3: ROC analysis for 60 lncRNA transcripts associated with ER+; Table S4: ROC analysis for 60 lncRNA transcripts associated with normal; Table S5: ROC analysis for 18 lncRNA transcripts associated with TNBC in validation cohort of 360 TNBC and 88 normal; and Table S6: ROC analysis for 18 lncRNA transcripts associated with TNBC in validation cohort of 360 TNBC and 88 normal. Figure S1: survival analysis of thirteen identified lncRNA transcripts in a cohort of 360 TNBC.
