# Supplementary material for: Molecular Classification of Breast Cancer Utilizing Long Non-Coding RNA (lncRNA) Transcriptomes Identifies Novel Diagnostic lncRNA Panel for Triple-Negative Breast Cancer
Source: Cancers (Basel). 2021 Oct 26;13(21):5350. doi: 10.3390/cancers13215350 (PMC8582428; doi:10.3390/cancers13215350)
Supplement: Supplementary file 1 [file cancers-13-05350-s001.zip › Supplementary figure S1.pdf]

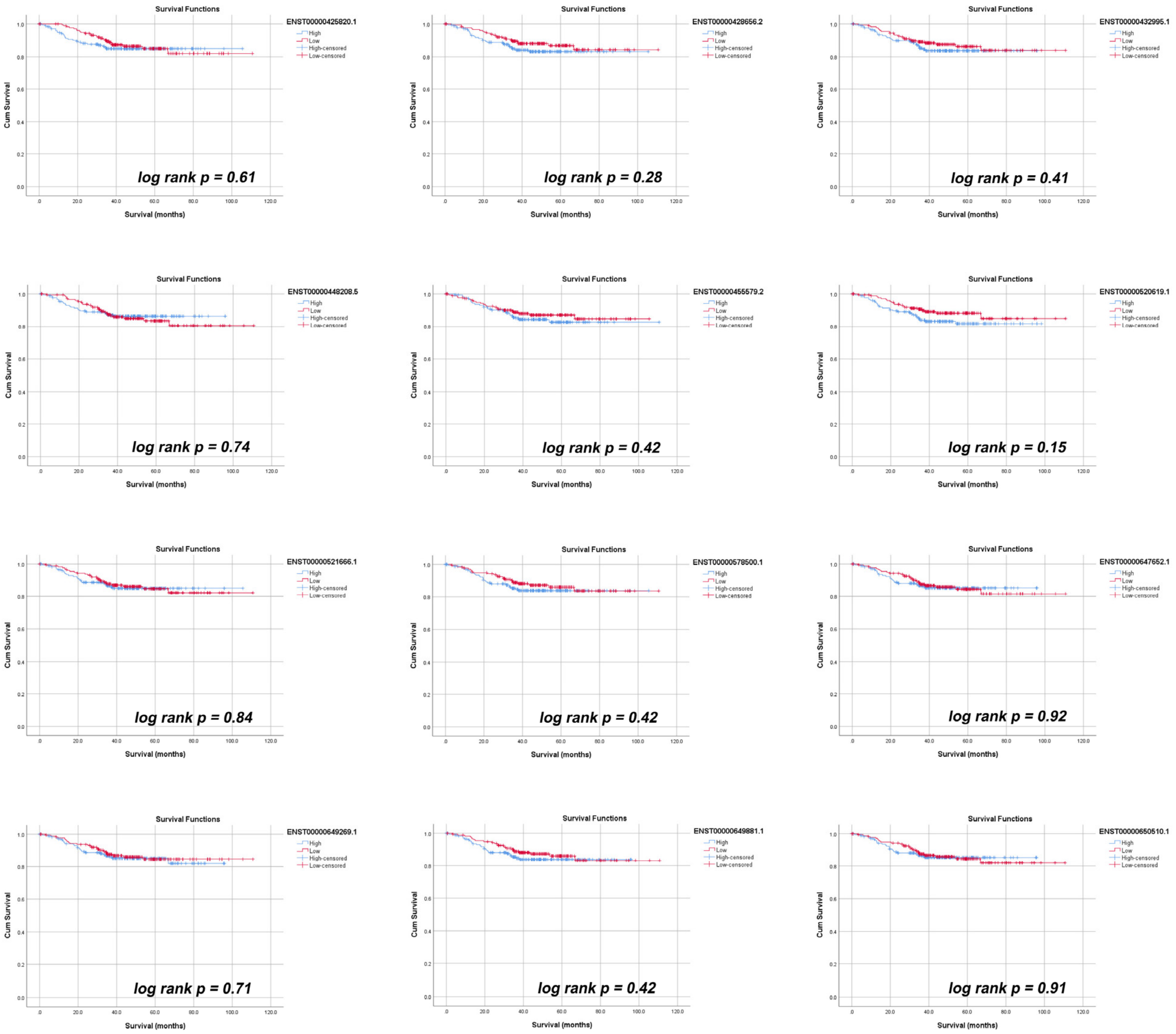


**Supplementary figure S1. Survival analysis of thirteen identified lncRNA transcripts in a cohort of 360 TNBC (PRJNA486023)**. The cohort was divided into High and Low based on median lncRNA expression and were subjected to Kaplan–Meier survival analysis in SPSS (v26). Log-rank tests p values are indicated on each plot.
